# How to Demonstrate the Association Between the Inflammation and Atrial Fibrillation Recurrence

**DOI:** 10.1002/clc.70177

**Published:** 2025-07-10

**Authors:** Naoya Kataoka, Teruhiko Imamura

**Affiliations:** ^1^ Second Department of Internal Medicine University of Toyama Toyama Japan

**Keywords:** ablation, C‐reactive protein, pulmonary vein isolation

To the Editor,

Previous studies have reported an association between atrial fibrillation (AF) and systemic inflammation. In the present study, the authors investigated the prognostic value of the CALLY index—a composite marker reflecting both systemic inflammation and nutritional status—in predicting AF recurrence following catheter ablation [1]. They demonstrated that the CALLY index was an independent predictor of post‐ablation AF recurrence. While this is an intriguing finding, several concerns merit consideration.

The authors' hypothesis is based on the assumption that systemic inflammation, as represented by the CALLY index, contributes to the development of AF [1]. However, the mechanisms underlying AF recurrence may differ from those responsible for the initial onset of AF. In particular, pulmonary vein reconnection is widely recognized as a primary cause of AF recurrence after ablation [2]. Did the authors assess the presence of pulmonary vein reconnection in patients with recurrent AF? It is possible that the CALLY index is more closely associated with non‐pulmonary vein foci of AF recurrence [3].

As the CALLY index includes albumin and C‐reactive protein levels, it may reflect hepatic function rather than cardiac‐specific inflammation. The direct relationship between the CALLY index and cardiac pathology thus remains unclear. Advanced imaging modalities—such as cardiac magnetic resonance imaging or computed tomography—may help elucidate the relationship among the CALLY index, myocardial inflammation, and AF recurrence.

How the CALLY index could be used to guide strategies for preventing AF recurrence remains uncertain. If the index reflects impaired hepatic function, interventions such as alcohol restriction might be beneficial [4]. However, in the present study, the prevalence of alcohol consumption did not differ significantly between patients with and without AF recurrence [1].

## Ethics Statement

The authors have nothing to report.

## Consent

The authors have nothing to report.

## Conflicts of Interest

The authors declare no conflicts of interest.

## Data Availability

The authors have nothing to report.
